# Supplementary material for: Hot spot prediction in protein-protein interactions by an ensemble system
Source: BMC Syst Biol. 2018 Dec 31;12(Suppl 9):132. doi: 10.1186/s12918-018-0665-8 (PMC6311905; doi:10.1186/s12918-018-0665-8)
Supplement: Supplementary file 2 — All relevant MATLAB code. A simple Matlab implement of our predictor. (ZIP 19,742 kb) [file 12918_2018_665_MOESM2_ESM.zip › Additional file 2/Read ASA/ASA website/dbMPIKT/1F47B.html]

 NetSurfP - Protein Surface Accessibility and Secondary Structure Predictions

  

|  |  |  |
| --- | --- | --- |
|  |  | NetSurfP - Protein Surface Accessibility and Secondary Structure Predictions  Technical University of Denmark |

  


---

```
# For publication of results, please cite:
# A generic method for assignment of reliability scores applied to solvent accessibility predictions.
# Bent Petersen, Thomas Nordahl Petersen, Pernille Andersen, Morten Nielsen and Claus Lundegaard
# BMC Structural Biology 2009, 9:51 doi:10.1186/1472-6807-9-51
#
# Column 1: Class assignment - B for buried or E for Exposed - Threshold: 25% exposure, but not based on RSA
# Column 2: Amino acid
# Column 3: Sequence name
# Column 4: Amino acid number
# Column 5: Relative Surface Accessibility - RSA
# Column 6: Absolute Surface Accessibility
# Column 7: Z-fit score for RSA prediction
# Column 8: Probability for Alpha-Helix
# Column 9: Probability for Beta-strand
# Column 10: Probability for Coil
E M  1F47B                  1    0.787 157.519  -1.354   0.003   0.003   0.994
E D  1F47B                  2    0.778 112.139  -0.891   0.018   0.019   0.964
E K  1F47B                  3    0.663 136.338   0.803   0.018   0.047   0.935
E P  1F47B                  4    0.509  72.270  -0.751   0.053   0.043   0.903
E K  1F47B                  5    0.594 122.248   0.615   0.113   0.043   0.844
E R  1F47B                  6    0.611 139.827   1.244   0.053   0.043   0.903
E K  1F47B                  7    0.402  82.733   0.816   0.053   0.043   0.903
E E  1F47B                  8    0.598 104.523   0.394   0.018   0.088   0.893
E A  1F47B                  9    0.343  37.799   1.048   0.005   0.262   0.733
B V  1F47B                 10    0.056   8.576   1.128   0.002   0.816   0.182
B I  1F47B                 11    0.031   5.754   0.562   0.001   0.959   0.040
B I  1F47B                 12    0.037   6.771  -0.332   0.001   0.959   0.040
B M  1F47B                 13    0.015   2.901   1.466   0.000   0.983   0.017
B N  1F47B                 14    0.044   6.486   0.734   0.001   0.959   0.040
B V  1F47B                 15    0.018   2.751   1.287   0.001   0.900   0.099
B A  1F47B                 16    0.060   6.601   0.704   0.002   0.816   0.182
B A  1F47B                 17    0.029   3.152  -0.015   0.004   0.420   0.576
E H  1F47B                 18    0.303  55.170   0.665   0.005   0.045   0.951
E H  1F47B                 19    0.645 117.271  -0.189   0.016   0.005   0.979
E G  1F47B                 20    0.724  56.955  -1.409   0.016   0.005   0.979
E S  1F47B                 21    0.420  49.189  -0.375   0.018   0.019   0.964
E E  1F47B                 22    0.455  79.541  -0.008   0.019   0.141   0.840
B L  1F47B                 23    0.051   9.320  -0.183   0.005   0.336   0.660
E N  1F47B                 24    0.345  50.479   1.126   0.064   0.216   0.721
B G  1F47B                 25    0.071   5.564  -0.996   0.879   0.010   0.111
E E  1F47B                 26    0.448  78.231   1.206   0.923   0.002   0.076
E L  1F47B                 27    0.368  67.308   1.871   0.970   0.001   0.030
B L  1F47B                 28    0.023   4.193   1.017   0.975   0.003   0.022
B L  1F47B                 29    0.151  27.740   1.490   0.970   0.001   0.030
E N  1F47B                 30    0.429  62.820   1.796   0.970   0.001   0.030
B S  1F47B                 31    0.050   5.883   0.947   0.970   0.001   0.030
B I  1F47B                 32    0.030   5.513   0.200   0.970   0.001   0.030
E Q  1F47B                 33    0.367  65.546   0.720   0.970   0.001   0.030
E Q  1F47B                 34    0.606 108.321   0.659   0.858   0.002   0.139
B A  1F47B                 35    0.205  22.635   0.339   0.246   0.004   0.750
E G  1F47B                 36    0.481  37.847  -0.362   0.016   0.005   0.979
B F  1F47B                 37    0.063  12.644   0.535   0.005   0.045   0.951
E I  1F47B                 38    0.369  68.173   1.546   0.022   0.359   0.619
B F  1F47B                 39    0.163  32.754   0.888   0.021   0.451   0.528
B G  1F47B                 40    0.196  15.417  -0.162   0.019   0.141   0.840
E D  1F47B                 41    0.463  66.646  -0.737   0.053   0.043   0.903
E M  1F47B                 42    0.373  74.577  -1.197   0.058   0.017   0.925
E N  1F47B                 43    0.314  45.999  -1.061   0.053   0.043   0.903
B I  1F47B                 44    0.045   8.417  -0.015   0.118   0.150   0.732
B Y  1F47B                 45    0.071  15.130  -0.037   0.148   0.418   0.435
B H  1F47B                 46    0.106  19.263   0.713   0.159   0.516   0.325
B R  1F47B                 47    0.134  30.594   0.930   0.159   0.516   0.325
B H  1F47B                 48    0.149  27.194   0.745   0.148   0.418   0.435
B L  1F47B                 49    0.143  26.257  -0.470   0.066   0.296   0.638
E S  1F47B                 50    0.332  38.922   0.176   0.064   0.216   0.721
E P  1F47B                 51    0.423  60.080  -1.308   0.052   0.084   0.864
E D  1F47B                 52    0.421  60.695  -1.446   0.018   0.047   0.935
E G  1F47B                 53    0.603  47.472  -2.453   0.018   0.019   0.964
E S  1F47B                 54    0.632  74.117  -1.146   0.018   0.019   0.964
E G  1F47B                 55    0.315  24.822  -0.647   0.018   0.047   0.935
E P  1F47B                 56    0.315  44.698   0.819   0.021   0.279   0.699
B A  1F47B                 57    0.057   6.314   0.275   0.018   0.846   0.136
B L  1F47B                 58    0.059  10.730   0.551   0.011   0.918   0.071
B F  1F47B                 59    0.026   5.298   0.839   0.006   0.962   0.032
B S  1F47B                 60    0.040   4.653  -0.390   0.006   0.962   0.032
B L  1F47B                 61    0.022   4.065   0.834   0.018   0.846   0.136
B A  1F47B                 62    0.030   3.350   0.146   0.023   0.655   0.322
B N  1F47B                 63    0.076  11.126  -0.706   0.148   0.418   0.435
B M  1F47B                 64    0.039   7.824  -0.482   0.118   0.150   0.732
B V  1F47B                 65    0.179  27.589  -0.881   0.113   0.087   0.800
E K  1F47B                 66    0.327  67.264   0.226   0.052   0.084   0.864
E P  1F47B                 67    0.287  40.697  -1.302   0.018   0.047   0.935
B G  1F47B                 68    0.158  12.466  -2.404   0.018   0.047   0.935
E T  1F47B                 69    0.326  45.272  -0.645   0.005   0.045   0.951
B F  1F47B                 70    0.073  14.691  -0.617   0.018   0.088   0.893
E D  1F47B                 71    0.432  62.309   0.688   0.018   0.047   0.935
B P  1F47B                 72    0.279  39.604  -0.422   0.018   0.047   0.935
E E  1F47B                 73    0.469  81.934   0.702   0.018   0.047   0.935
B M  1F47B                 74    0.127  25.313   0.472   0.053   0.043   0.903
E K  1F47B                 75    0.663 136.297   0.347   0.018   0.047   0.935
E D  1F47B                 76    0.587  84.601  -0.149   0.018   0.047   0.935
B F  1F47B                 77    0.130  26.131   0.079   0.019   0.141   0.840
E T  1F47B                 78    0.479  66.437  -0.393   0.021   0.279   0.699
B T  1F47B                 79    0.134  18.530   0.054   0.005   0.262   0.733
E P  1F47B                 80    0.342  48.544   0.717   0.004   0.197   0.799
B G  1F47B                 81    0.061   4.801  -0.272   0.004   0.514   0.481
B V  1F47B                 82    0.029   4.457   0.719   0.001   0.900   0.099
B T  1F47B                 83    0.037   5.076   0.315   0.001   0.959   0.040
B I  1F47B                 84    0.022   4.033   1.219   0.001   0.959   0.040
B F  1F47B                 85    0.027   5.519   0.638   0.001   0.959   0.040
B M  1F47B                 86    0.029   5.883   0.118   0.001   0.900   0.099
B Q  1F47B                 87    0.118  21.111  -0.567   0.003   0.718   0.279
B V  1F47B                 88    0.036   5.456   0.452   0.004   0.197   0.799
B P  1F47B                 89    0.188  26.748  -0.034   0.005   0.045   0.951
E S  1F47B                 90    0.300  35.125   0.068   0.005   0.015   0.979
B Y  1F47B                 91    0.256  54.729  -0.254   0.018   0.019   0.964
E G  1F47B                 92    0.575  45.221  -1.485   0.053   0.005   0.942
E D  1F47B                 93    0.435  62.684   0.340   0.109   0.005   0.886
B E  1F47B                 94    0.048   8.333  -2.200   0.782   0.003   0.216
E L  1F47B                 95    0.304  55.699   0.262   0.858   0.002   0.139
E Q  1F47B                 96    0.392  70.047   1.284   0.923   0.002   0.076
B L  1F47B                 97    0.037   6.738  -0.752   0.970   0.001   0.030
B F  1F47B                 98    0.027   5.479   0.790   0.970   0.001   0.030
E K  1F47B                 99    0.389  80.058   1.015   0.970   0.001   0.030
B L  1F47B                100    0.218  40.007   1.330   0.970   0.001   0.030
B M  1F47B                101    0.018   3.602   1.071   0.988   0.000   0.012
B L  1F47B                102    0.067  12.268   0.857   0.988   0.000   0.012
E Q  1F47B                103    0.477  85.282   1.147   0.988   0.000   0.012
B S  1F47B                104    0.070   8.263   0.491   0.988   0.000   0.012
B A  1F47B                105    0.014   1.543   1.207   0.988   0.000   0.012
E Q  1F47B                106    0.288  51.365   1.249   0.970   0.001   0.030
E H  1F47B                107    0.353  64.247   1.881   0.970   0.001   0.030
B I  1F47B                108    0.027   5.051   0.881   0.970   0.001   0.030
B A  1F47B                109    0.029   3.240  -0.224   0.970   0.001   0.030
E D  1F47B                110    0.652  94.025   1.353   0.938   0.007   0.055
E E  1F47B                111    0.433  75.628   0.811   0.782   0.003   0.216
B V  1F47B                112    0.071  10.990   0.494   0.406   0.004   0.590
E G  1F47B                113    0.600  47.228   0.634   0.016   0.005   0.979
B G  1F47B                114    0.068   5.383  -0.649   0.019   0.141   0.840
E V  1F47B                115    0.333  51.121   1.255   0.002   0.816   0.182
B V  1F47B                116    0.057   8.807   0.780   0.001   0.900   0.099
B L  1F47B                117    0.180  32.976   1.339   0.018   0.846   0.136
B D  1F47B                118    0.120  17.306  -0.192   0.021   0.451   0.528
E D  1F47B                119    0.478  68.937  -0.855   0.018   0.047   0.935
E Q  1F47B                120    0.569 101.623  -0.154   0.018   0.019   0.964
B R  1F47B                121    0.304  69.593   0.154   0.018   0.019   0.964
E R  1F47B                122    0.421  96.478   0.019   0.018   0.047   0.935
E M  1F47B                123    0.427  85.523   0.377   0.018   0.047   0.935
B M  1F47B                124    0.071  14.247   0.453   0.018   0.047   0.935
E T  1F47B                125    0.369  51.194   1.195   0.018   0.019   0.964
E P  1F47B                126    0.576  81.720   0.941   0.923   0.002   0.076
E Q  1F47B                127    0.662 118.233   0.508   0.923   0.002   0.076
B K  1F47B                128    0.189  38.816   0.899   0.970   0.001   0.030
B L  1F47B                129    0.129  23.675   0.502   0.988   0.000   0.012
E R  1F47B                130    0.515 117.866   1.492   0.970   0.001   0.030
E E  1F47B                131    0.448  78.213   1.929   0.970   0.001   0.030
B Y  1F47B                132    0.040   8.612   0.767   0.970   0.001   0.030
B Q  1F47B                133    0.243  43.436   1.374   0.988   0.000   0.012
E D  1F47B                134    0.571  82.209   1.463   0.988   0.000   0.012
B I  1F47B                135    0.253  46.750   1.868   0.988   0.000   0.012
B I  1F47B                136    0.023   4.347   1.144   0.988   0.000   0.012
E R  1F47B                137    0.400  91.577   1.794   0.970   0.001   0.030
E E  1F47B                138    0.475  82.895   1.717   0.970   0.001   0.030
B V  1F47B                139    0.108  16.661   1.398   0.923   0.002   0.076
E K  1F47B                140    0.348  71.625   0.842   0.923   0.002   0.076
E D  1F47B                141    0.618  89.039   0.415   0.858   0.002   0.139
E A  1F47B                142    0.658  72.468   0.201   0.600   0.003   0.397
E N  1F47B                143    0.506  74.137  -0.713   0.246   0.004   0.750
E A  1F47B                144    0.833  91.819  -0.514   0.003   0.003   0.994

Explain the output.  Go back.
```

---
